# Supplementary material for: Noninvasive optical activation of Flp recombinase for genetic manipulation in deep mouse brain regions
Source: Nat Commun. 2019 Jan 18;10:314. doi: 10.1038/s41467-018-08282-8 (PMC6338782; doi:10.1038/s41467-018-08282-8)
Supplement: Supplementary file 1 — Supplementary Information [file 41467_2018_8282_MOESM1_ESM.pdf]

Supplementary Information

Noninvasive optical activation of Flp recombinase

for genetic manipulation in deep mouse brain regions

Jung et al.

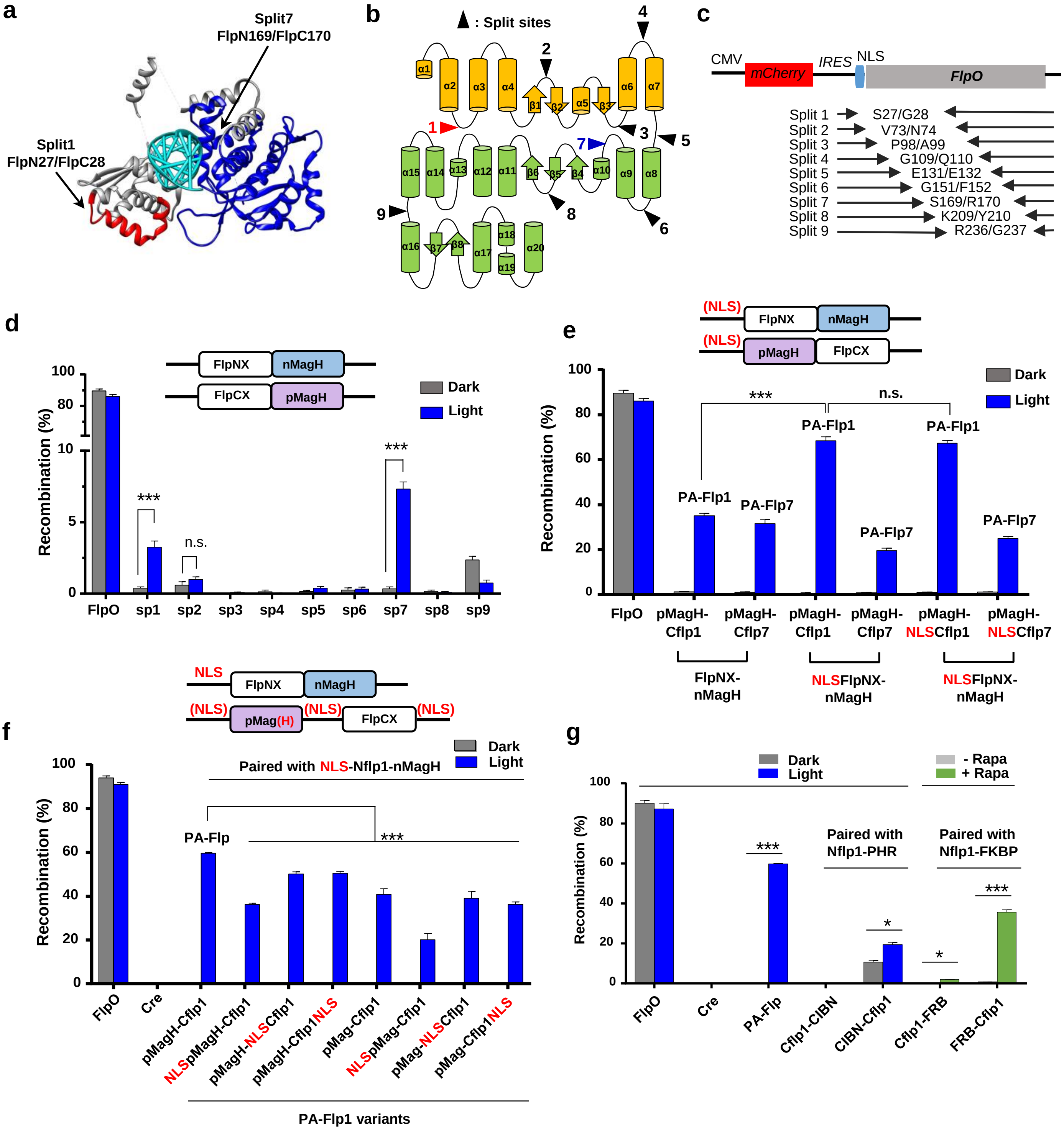

a
Split7
FlpN169/FlpC170
Split1
FlpN27/FlpC28
b
: Split sites
c
CMV
mCherry
IRES
NLS
FlpO
Split 1 S27/G28
Split 2 V73/N74
Split 3 P98/A99
Split 4 G109/Q110
Split 5 E131/E132
Split 6 G151/F152
Split 7 S169/R170
Split 8 K209/Y210
Split 9 R236/G237
d
FlpNX
nMagH
FlpCX
pMagH
Dark
Light
Recombination (%)
FlpO sp1 sp2 sp3 sp4 sp5 sp6 sp7 sp8 sp9
e
(NLS)
pMagH-Cflp1
pMagH-Cflp7
pMagH-NLSCflp1
pMagH-NLSCflp7
FlpNX-nMagH
NLSFlpNX-nMagH
PA-Flp1
PA-Flp7
f
NLS
pMag(H)
Paired with NLS-Nflp1-nMagH
PA-Flp
FlpO
Cre
pMagH-Cflp1
NLSpMagH-Cflp1
pMagH-NLSCflp1
pMagH-Cflp1NLS
pMag-Cflp1
NLSpMag-Cflp1
pMag-NLSCflp1
pMag-Cflp1NLS
PA-Flp1 variants
g
- Rapa
+ Rapa
Paired with Nflp1-PHR
Paired with Nflp1-FKBP
Cflp1-CIBN
CIBN-Cflp1
Cflp1-FRB
FRB-Cflp1

Supplementary Figure 1. Screening procedures used to develop the highly efficient split-Flp–based PA-Flp system.

(a) Ribbon representation of Flp (PDB 1M6X). Arrows indicate the split sites for split-1 (S27/G28) and split-7 (S169/R170). **(b)** The N-terminal domain (yellow) or C-terminal domain (green) of Flp recombinase are colored. All nine tested split sites on Flp are marked by arrows. **(c)** Screening plasmid platform and exact split positions of each split-Flp construct. **(d–g)** Recombination efficiency measured in HEK293T cells using a Flp reporter (Floxed-STOP-GFP) on a blue LED array. Data represent means ±s.e.m. (n = 3 trials). **(d)** Reconstitution efficiency (percentage) of the nine split-site variants screened. N- and C-terminal fragments of Flp were each fused at their N-terminus with nMagHigh and pMagHigh, respectively. Split-site variants 1 (sp1) and 7 (sp7), which showed a significant increase in recombination efficiency, were selected as final PA-Flp candidates. **(e)** Comparison of recombination efficiency among PA-Flp1 and PA-Flp7 variants indicated, with or without an NLS tag on FlpNX-nMagH. **(f)** Additional optimization of PA-Flp1 variants, by tagging NLS or not on indicated sites of pMagH-Cflp1. The final PA-Flp1 variants selected (NLS-Nflp1-nMagH and pMagH-Cflp1 pair) is denoted PA-Flp. **(g)** Comparison of Magnet-based PA-Flp with CRY2-CIBN– and FRB-FKBP–based split-Flp1 systems, designed on the same platform as PA-Flp. +Rapa : Rapamycin (100 nM) treatment, -Rapa : Vehicle (0.1% Dimethyl Sulfoxide) treatment.

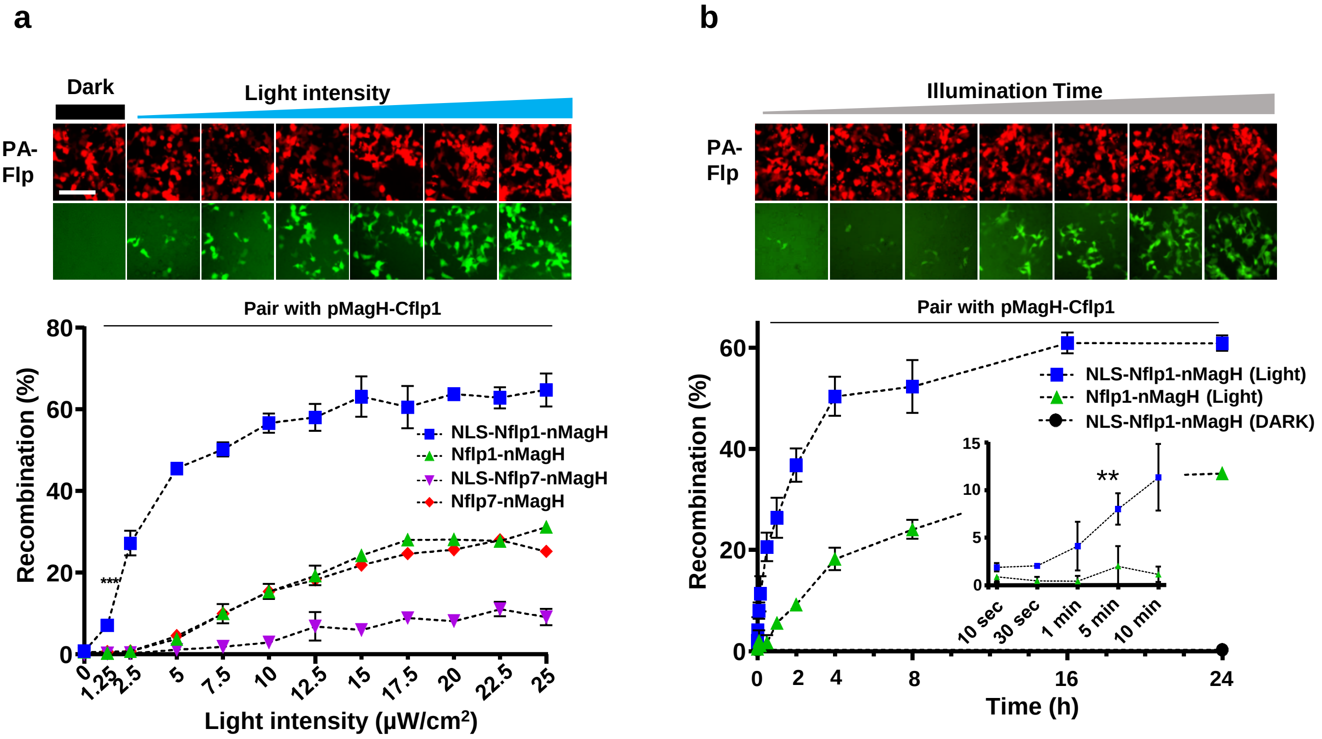

Supplementary Figure 2. Characterization of the light sensitivity of PA-Flp.

(a, b) Recombination efficiency of PA-Flp1 or 7 variants according to light intensity **(a)** and duration **(b)**. Recombination efficiency was measured in HEK293T cells co-transfected with the indicated PA-Flp1 or 7 variants and Floxed-STOP-GFP and stimulated using a blue LED array. Error bars: ± s.e.m. (n = 3 trials). **(a)** Transfected cells were illuminated by blue light (1.25–25 μW cm^{-2}) on a 10-s ON/3-min OFF cycle for 24 h. **(b)** Transfected cells were illuminated by blue light for the indicated durations (0, 1, 2, 4, 8, 16, and 24 h) with 10-s ON/3-min OFF cycle at a fixed intensity of 10 μW cm^{-2}. Scale bar: 100 μm.

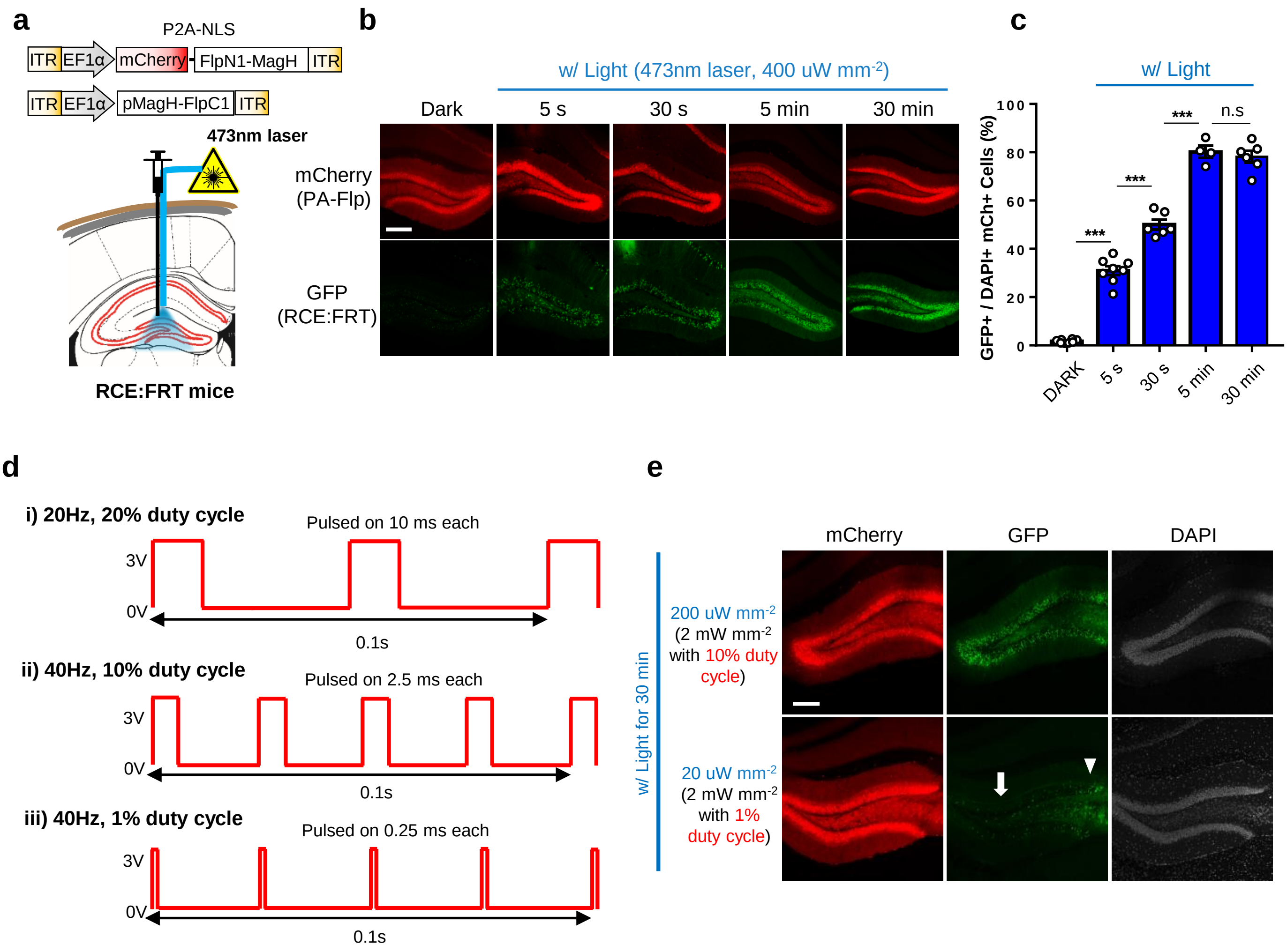

Supplementary Figure 3. Characterization of PA-Flp activity in the mouse brain according to light-delivery conditions.

(a) Schematic depicting AAV-EF1a-PA-Flp targeting in the hippocampal DG of 8-wk-old mice, followed by light stimulation via an implanted optic fiber. **(b)** PA-Flp (mCherry) and Flp reporter (GFP) signal intensities according to different light stimulation times at the same blue laser power (400 μW mm^{-2} at optic fiber output). w/ Light: with Light. Scale bar: 200 μm. **(c)** Measurement of GFP positive cells among both DAPI and mCherry positive (GFP+/DAPI+mCh+) cells at each stimulation time in 4-8 coronal slices, as shown in **b**. Data represent means ± s.e.m. (n = 2 mice/group, ****P* < 0.0001). **(d)** Examples of controlling pulse frequency (Hz) or duty cycle (%) for precise adjustment of light delivery. **(e)** Representative images showing the effect of local light delivery depending on duty cycle adjustments with the same light intensity at the optic fiber tip (Ø 50 μm, 20 μW mm^{-2} output). Arrowhead indicates illuminated regions; arrow indicates a region unaffected by light delivery. Scale bar: 200 μm.

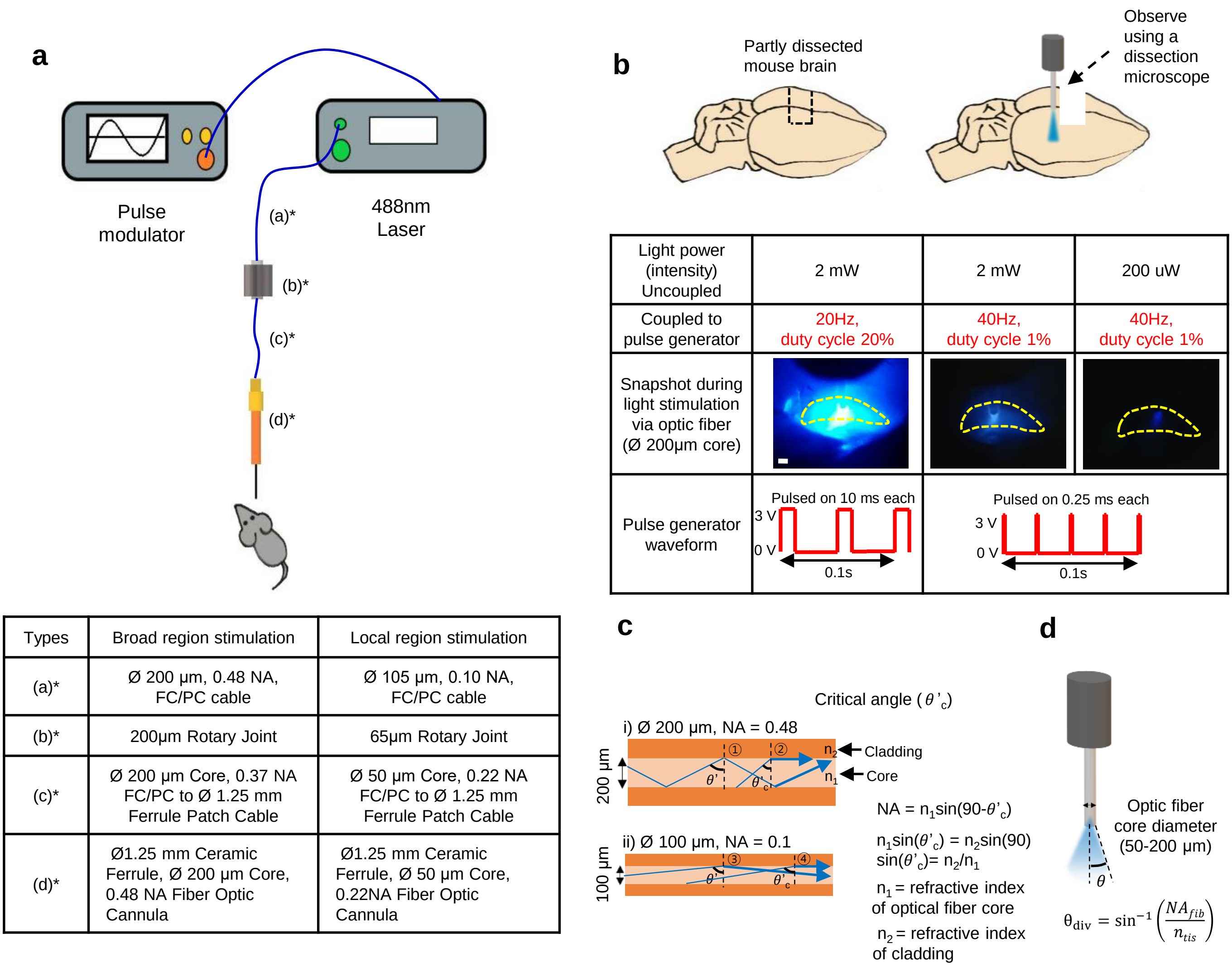

Light power (intensity) Uncoupled	2 mW	2 mW	200 uW
Coupled to pulse generator	20Hz, duty cycle 20%	40Hz, duty cycle 1%	40Hz, duty cycle 1%
Snapshot during light stimulation via optic fiber (Ø 200µm core)			
Pulse generator waveform	Pulsed on 10 ms each	Pulsed on 0.25 ms each	

Types	Broad region stimulation	Local region stimulation
(a)*	Ø 200 µm, 0.48 NA, FC/PC cable	Ø 105 µm, 0.10 NA, FC/PC cable
(b)*	200µm Rotary Joint	65µm Rotary Joint
(c)*	Ø 200 µm Core, 0.37 NA FC/PC to Ø 1.25 mm Ferrule Patch Cable	Ø 50 µm Core, 0.22 NA FC/PC to Ø 1.25 mm Ferrule Patch Cable
(d)*	Ø1.25 mm Ceramic Ferrule, Ø 200 µm Core, 0.48 NA Fiber Optic Cannula	Ø1.25 mm Ceramic Ferrule, Ø 50 µm Core, 0.22NA Fiber Optic Cannula

Supplementary Figure 4. Optical settings for light delivery in local regions of the mouse brain.

(a) Schematic depicting optical settings for light stimulation of broad versus local regions using a 473-nm laser-optic fiber. **(b)** Light diffusion and scattering patterns at each laser power, frequency, and duty cycle were monitored in the brain of an anesthetized mouse via a camera connected to a dissecting microscope. Dashed black lines indicate dissected planes of the mouse brain. Scale bar: 200 µm. **(c)** A 200-µm core patch cord with a high numerical aperture (NA=0.48) or 100-µm core patch cord with low NA (0.1) were used to obtain an appropriate light diffusion pattern—broad or local—within a region of brain tissue at the optic fiber tip. **(d)** Fiber optic ferrule. The optic fiber core diameter (Ø 50–200 µm) is denoted by an arrow. The diffusion angle of light at the optic fiber tip can be estimated from the following equation: $\theta_{div} = \sin^{-1}(NA_{fib}/n_{tis})$, where θ_{div} is the half-angle of divergence, NA_{fib} is the the numerical aperture of the optic fiber, and n_{tis} is the refractive index of gray matter.

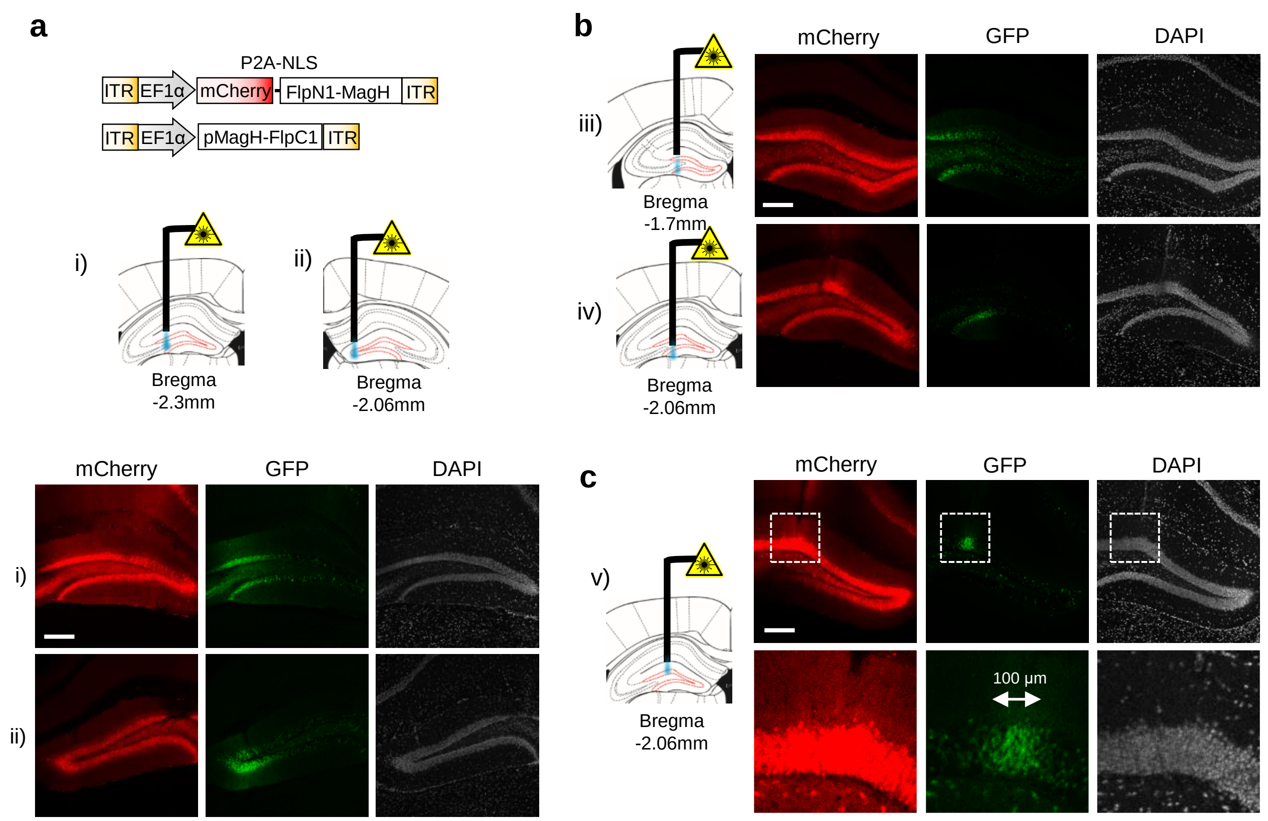

Supplementary Figure 5. Light-inducible genetic labeling with diverse localized light patterns within the hippocampal DG.

(a–c) AAV-EF1a-PA-Flp targeting in the hippocampal DG of 8–12-wk-old mice, followed by optic fiber ferrule implantation in the indicated regions. Light was delivered using a 473-nm laser-coupled thin optic fiber (Ø 50 µm) with a NA=0.48 FC/PC cable **(a, b)** or NA=0.1 FC/PC cable (see Supplementary Figure 4a, (a)*–(d)*). **(c)** Light was delivered with 5 µW mm^{-2} for 2 h **(a)** or 30 min **(b)**; 1 µW mm^{-2} for 30 min **(c)**. Scale bar: 200 µm.

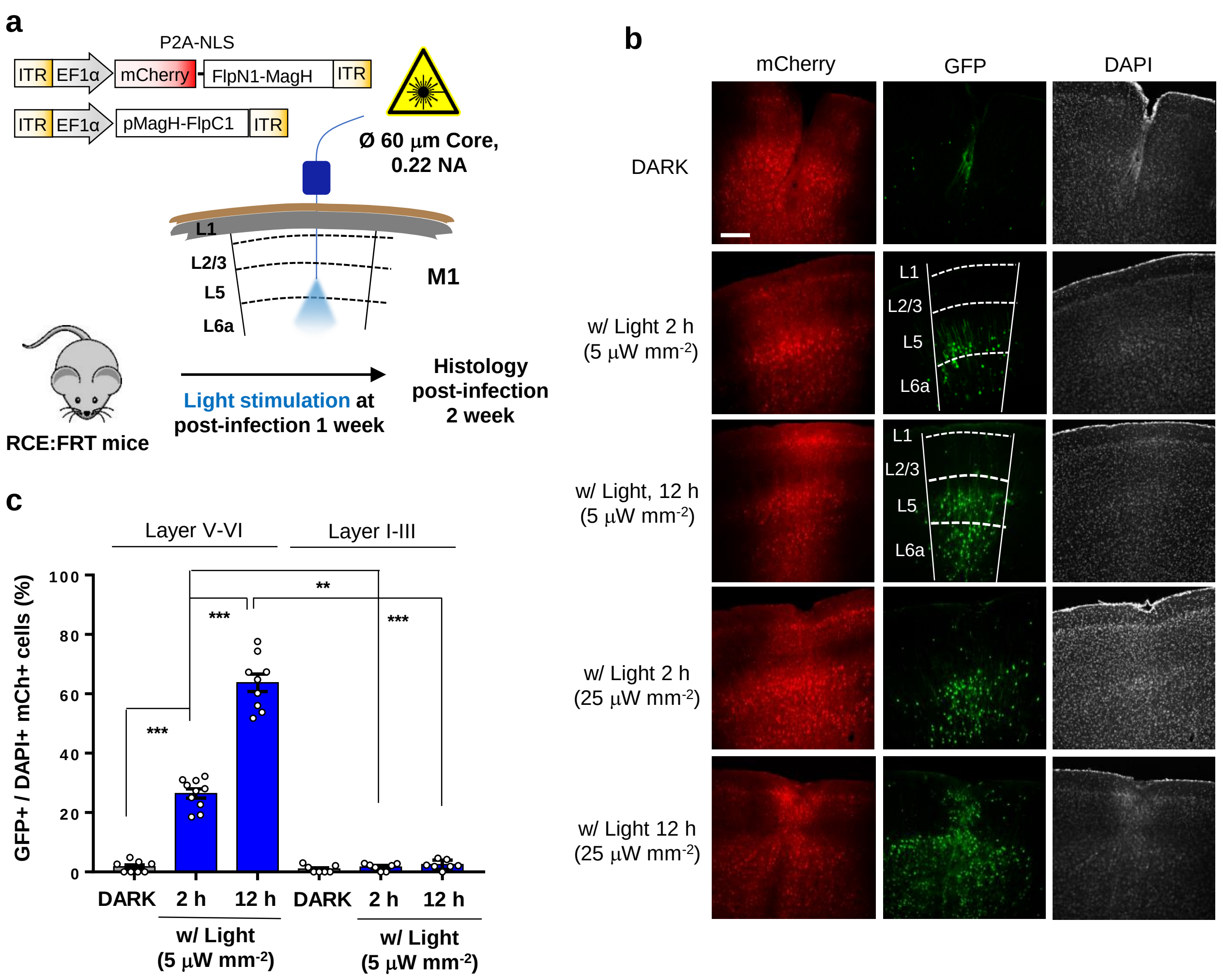

Supplementary Figure 6. Layer 5/6-specific genetic labeling in the cortex region.

(a) Schematic depicting AAV infection of 8–12-wk-old mice, and local light stimulation in layer 5/6 of the primary motor cortex (M1). **(b)** Infection of layer I-VI (L1-L6) with AAVs (5×10^8 viral genomes) expressing PA-Flp$_{\triangle NLS}$. Tiny optic fibers were implanted into layer V (DV 0.65) of the M1 cortex immediately after AAV infection. One week after infection, light was delivered at an intensity of 5 or 25 μW mm^{-2} for 2 or 12 h. w/ Light: with Light. **(c)** Quantification of cells positive for the Flp reporter signal (GFP) among DAPI and mCherry positive (GFP+/DAPI+mCh+) cells in 7-10 coronal slices at each group. All cells in layer V-VI or I-III were measured at a constant power of 5 μW mm^{-2}, delivered for 2 or 12 h. Data represent means ± s.e.m (n = 2 mice/group; ***$P < 0.0005$; **$P < 0.005$; two-tailed Student's t-test). Scale bar: 200 μm.

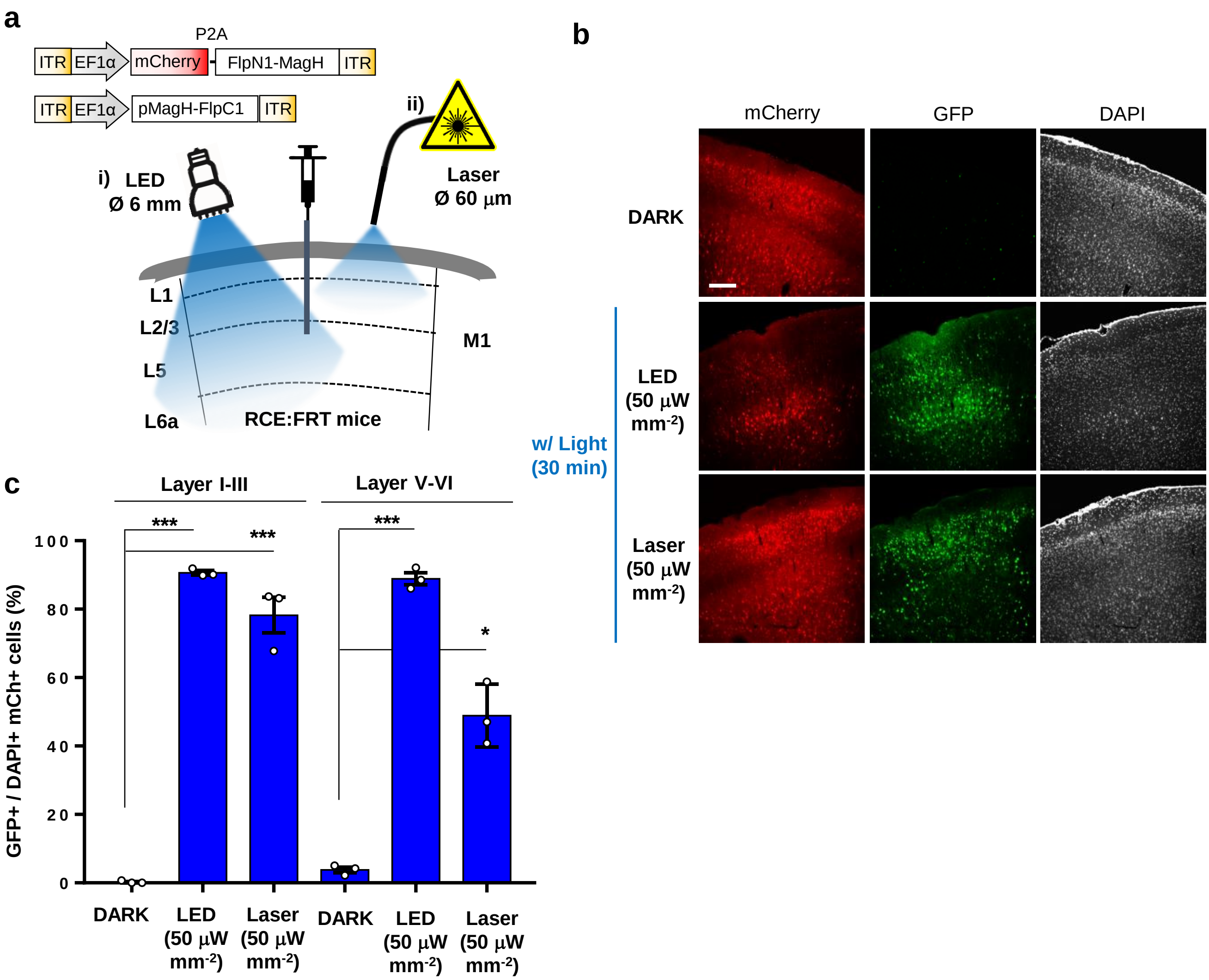

Supplementary Figure 7. Genetic labeling in primary motor cortex (M1) using noninvasive light delivery.

(a) Schematic depicting AAV infection of 8-wk-old mice, and noninvasiveasive light delivery in the M1 cortex via a white LED (Ø 6 mm) **i)** or laser-optic fiber (Ø 60 µm, 0.37 NA) **ii)**. **(b)** Infection across layers I-VI with AAVs expressing PA-Flp$_{\triangle NLS}$. A white LED (50 µW mm^{-2}) or 473-nm laser-optic fiber (50 µW mm^{-2}) was used to deliver light transcranially for 30 min in a dark room. Note that the core diameter of the LED (Ø 6 mm) is 100-times larger than that of the optic fiber (Ø 60 µm), but the light intensity per unit area (µW mm^{-2}) is the same. w/ Light: with Light. **(c)** Layer I-III or V-VI cells were assessed following 30-min light stimulation with a white LED or optic fiber (50 µW mm^{-2} for both). Quantification of cells positive for the Flp reporter signal (GFP) among DAPI and mCherry positive (GFP+/DAPI+mCh+) cells in 3 coronal slices at each group. The measurement was performed in each layer I-III or V-VI regions of 3 coronal slices. Data represent means ± s.e.m (n = 2 mice for dark, LED, and laser groups; ***$P < 5 \times 10^{-5}$, *$P < 0.05$, two-tailed Student's t-test). Scale bar: 200 µm.

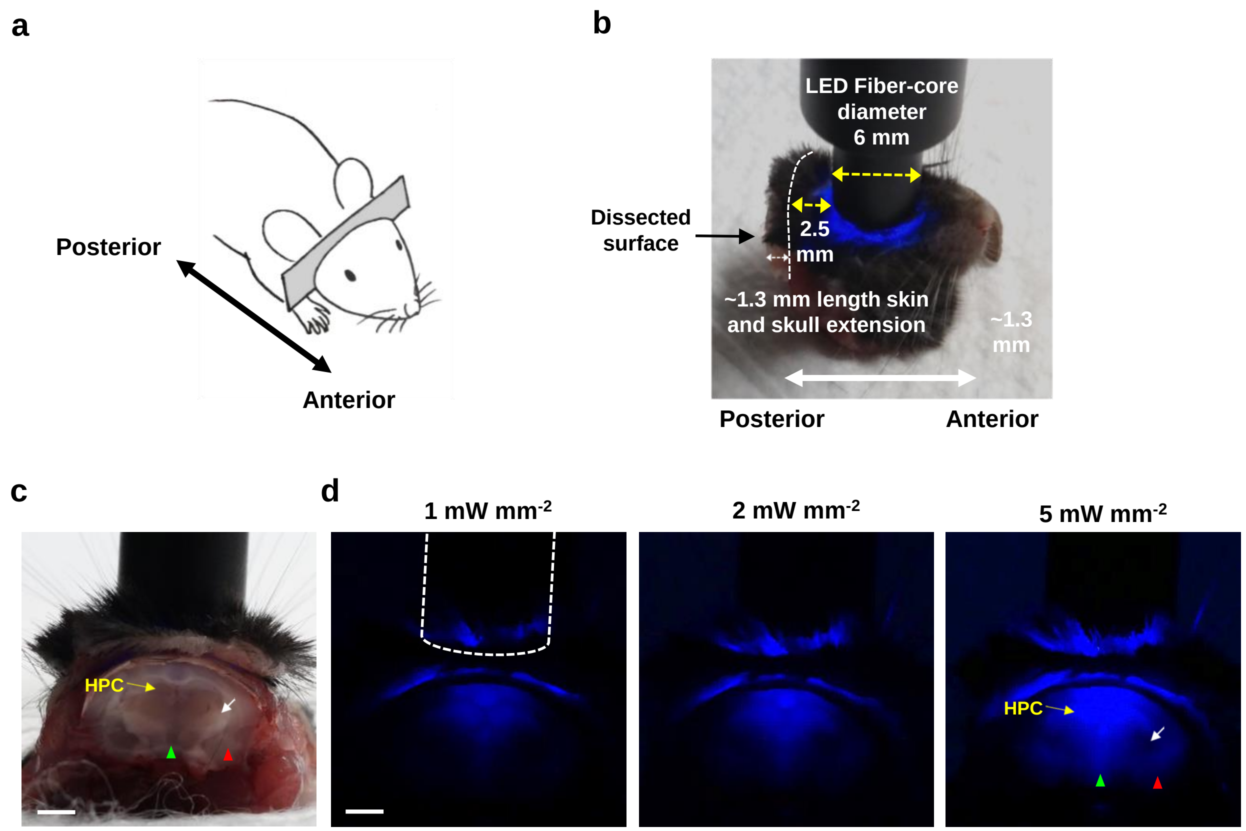

Supplementary Figure 8. Top-down, noninvasive LED penetration and scattering patterns in the mouse brain.

(a) Schematic depicting cutting of the mouse brain along a coronal plane (8-wk-old mice. **(b)** Side view of top-down, fiber-type blue LED (Ø 6 mm) illumination of the mouse brain. The light path was restricted from passing outside of the brain tissue by placing the fiber-type LED core at a distance of 2.5 mm from the dissected plane of the brain tissue. In addition, the skin and skull extended longer along the sagittal axis (~1.3 mm) than the dissected plane of brain tissue, thereby thoroughly excluding scattered light from occurring at the interface between the LED fiber core and skin. **(c, d)** Photos of a dissected mouse brain in relation to LED position during top-down illumination in a light **(c)** or dark **(d)** environment. In d, differences in blue light distribution in mouse brain tissue between white and gray matter produced by top-down, noninvasive LED illumination are shown. Note that even gray matter (green and red arrowhead) at a depth below white matter (white arrow) shows bright penetrating light owing to the higher refractive index of white matter compared with that of gray matter. White arrow, deep cerebral white matter; green arrowhead, hypothalamus; red arrowhead, amygdala; HPC: hippocampus. Photos were taken under the same imaging conditions (focal length and exposure time) at the indicated LED intensities. Scale bar: 2 mm.

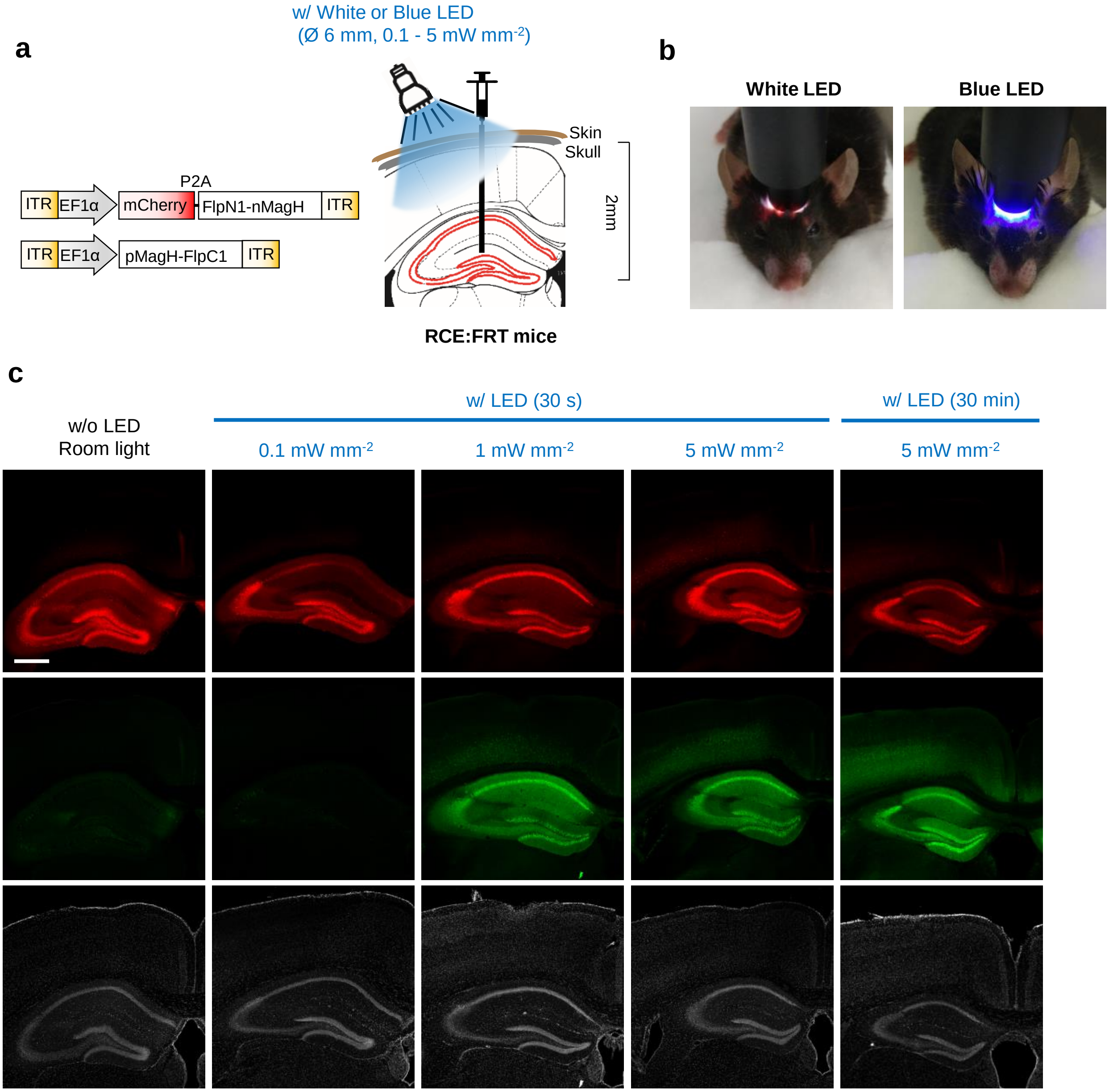

Supplementary Figure 9. Noninvasive activation of PA-Flp in the hippocampus according to light delivery conditions.

(a) Schematic depicting AAV targeting in the hippocampus of 8-wk-old mice, followed by white or blue LED illumination. **(b)** Photographs showing noninvasive white or blue light delivery via LED illumination of the anesthetized mouse brain. **(c)** Efficacy of PA-Flp activation in the hippocampus by noninvasive white LED illumination at the indicated intensities for 30-s. w/ LED: with LED, w/o LED: without LED in room light. Scale bar: 500 μm.

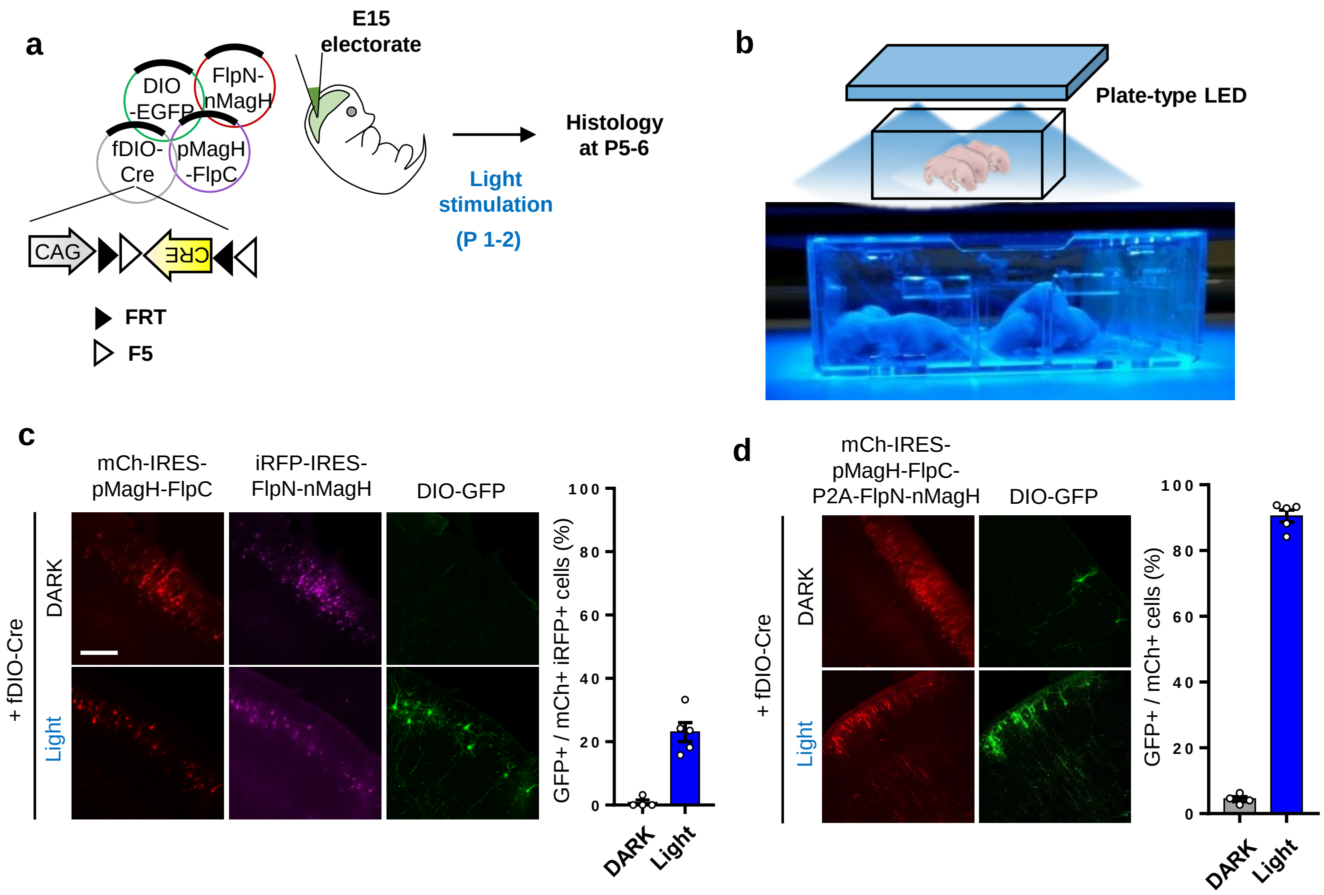

Supplementary Figure 10. Verification of the fDIO-Cre system by in vivo electroporation.

(a) Schematic depicting the design of fDIO-Cre and in vivo electroporation of the indicated plasmids. **(b)** Blue LED illumination using a plate-type LED. **(c, d)** CAG promoter-driven PA-Flp, fDIO-Cre, and DIO-GFP (Double-floxed Cre reporter) were co-expressed in the cortex. The expression of each split-Flp component was confirmed by monitoring mCherry and iRFP fluorescence **(c)**, or mCherry fluorescence **(d)**. Self-cleaving 2A peptide (P2A) or an IRES sequence was used to construct a PA-Flp dual-plasmid (mCherry-P2A (IRES)-pMagH-FlpC and iRFP682-P2A (IRES)-FlpN-nMagH) **(c)** or single-plasmid (mCherry-IRES-FlpN-nMagH-P2A-pMagH-FlpC) **(d)** bicistronic platform. Measurement of GFP positive cells among both mCherry and iRFP positive (GFP+/mCh+iRFP+) cells or among mCherry positive (GFP+/mCh+) cells in 4-6 coronal slices at each group. +fDIO-Cre: fDIO-Cre co-transfection. Data represent means ± s.e.m (n = 2 mice/group). Scale bar: 100 μm.

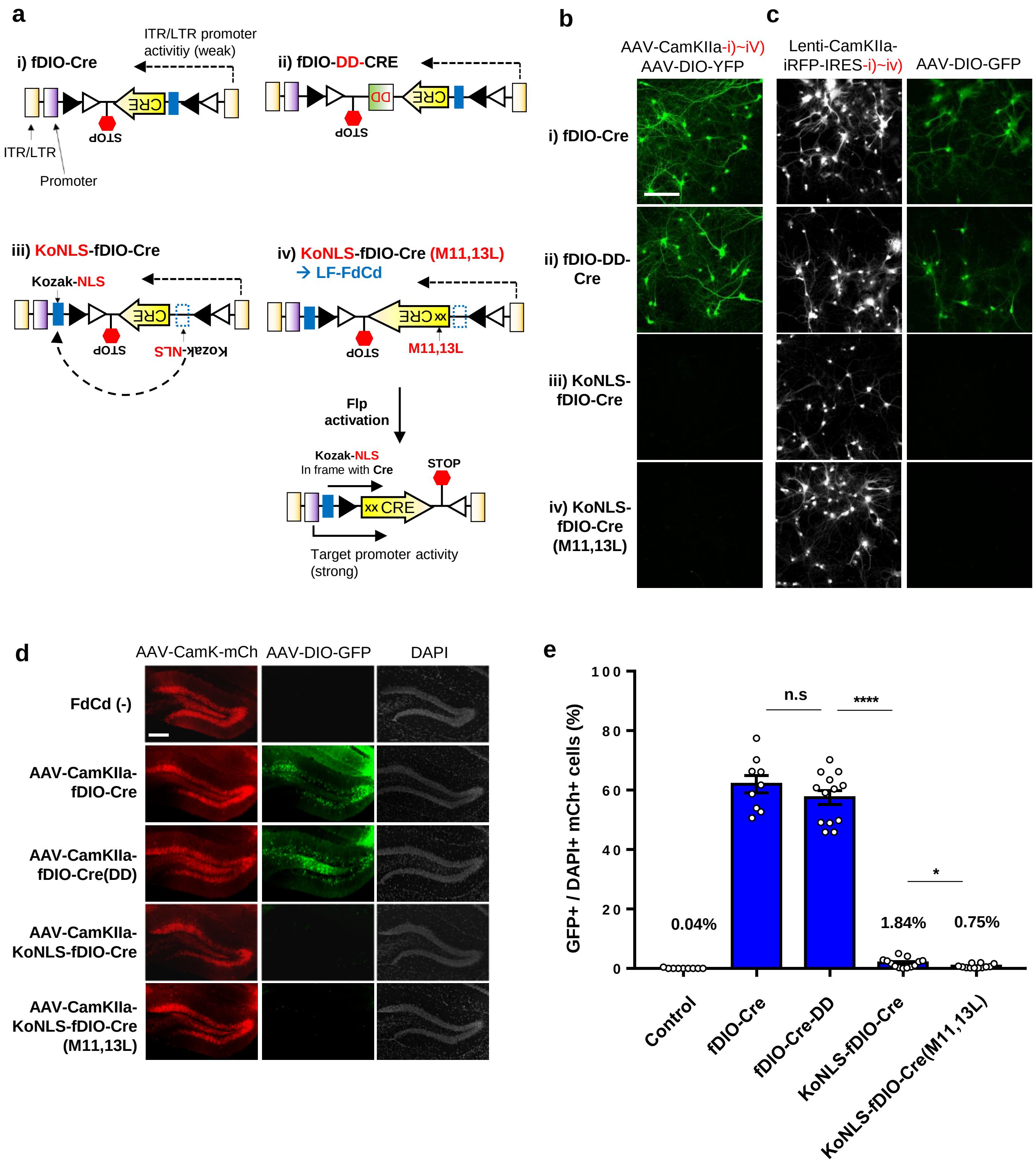
a
i) fDIO-Cre
ITR/LTR promoter activitiy (weak)
ITR/LTR
Promoter
STOP
CRE
ii) fDIO-DD-CRE
DD
iii) KoNLS-fDIO-Cre
Kozak-NLS
iv) KoNLS-fDIO-Cre (M11,13L)
→ LF-FdCd
M11,13L
Flp activation
Kozak-NLS
In frame with Cre
xx CRE
Target promoter activity (strong)
b
AAV-CamKIIa-i)~iV)
AAV-DIO-YFP
c
Lenti-CamKIIa-iRFP-IRES-i)~iv)
AAV-DIO-GFP
i) fDIO-Cre
ii) fDIO-DD-Cre
iii) KoNLS-fDIO-Cre
iv) KoNLS-fDIO-Cre (M11,13L)
d
AAV-CamK-mCh
AAV-DIO-GFP
DAPI
FdCd (-)
AAV-CamKIIa-fDIO-Cre
AAV-CamKIIa-fDIO-Cre(DD)
AAV-CamKIIa-KoNLS-fDIO-Cre
AAV-CamKIIa-KoNLS-fDIO-Cre (M11,13L)
e
GFP+ / DAPI+ mCh+ cells (%)
100
80
60
40
20
0
n.s
****
*
0.04%
1.84%
0.75%
Control
fDIO-Cre
fDIO-Cre-DD
KoNLS-fDIO-Cre
KoNLS-fDIO-Cre(M11,13L)

Supplementary Figure 11. Evaluation of Flp-dependent Cre driver (FdCd) candidates as a virus-mediated gene delivery system for blockade of leaky Cre expression.

(a) Schematic depicting designs of AAV and lentiviral vectors containing Flp-dependent Cre driver candidates. Codon-optimized Cre (iCre) is inserted into the double-flox–oriented sequence of a Flp recombinase (fDIO) cassette. Tagging Cre with a GFP destabilized domain (DD-Cre), and reorganizing NLS and Kozak sequence out of the fDIO cassette with subsequent dual-site mutation of N-terminal regions of Cre (M11L, M13L) are shown as candidates of Flp-dependent Cre-driver strategies. **(b, c)** Cultured hippocampal neurons were co-infected with AAV-EF1a-DIO-GFP (Cre reporter) and AAVs (multiplicity of infection [MOI] = 5) **(b)** or lentiviruses (MOI = 2–3) **(c)** carrying CamKIIa promoter-driven FdCd candidates shown in **a**. Scale bar: 200 μm. **(d)** Fluorescence images of the hippocampal DG of 8–12-wk-old WT mice co-infected with AAVs carrying Flp-dependent Cre candidates and the Cre reporter AAV-CAG-DIO-GFP (each at 5×10^8 viral genomes), obtained after incubating for 3 wk. **(e)** Quantitative analysis of results in **d**, showing percentages of GFP positive cells among mCherry (expression marker) positive cells. Data represent means ± s.e.m (n = 2 mice/group; $^{****}P < 1 \times 10^{-10}$, $^{*}P < 0.05$, two-tailed Student’s *t*-test). Scale bar: 200 μm.

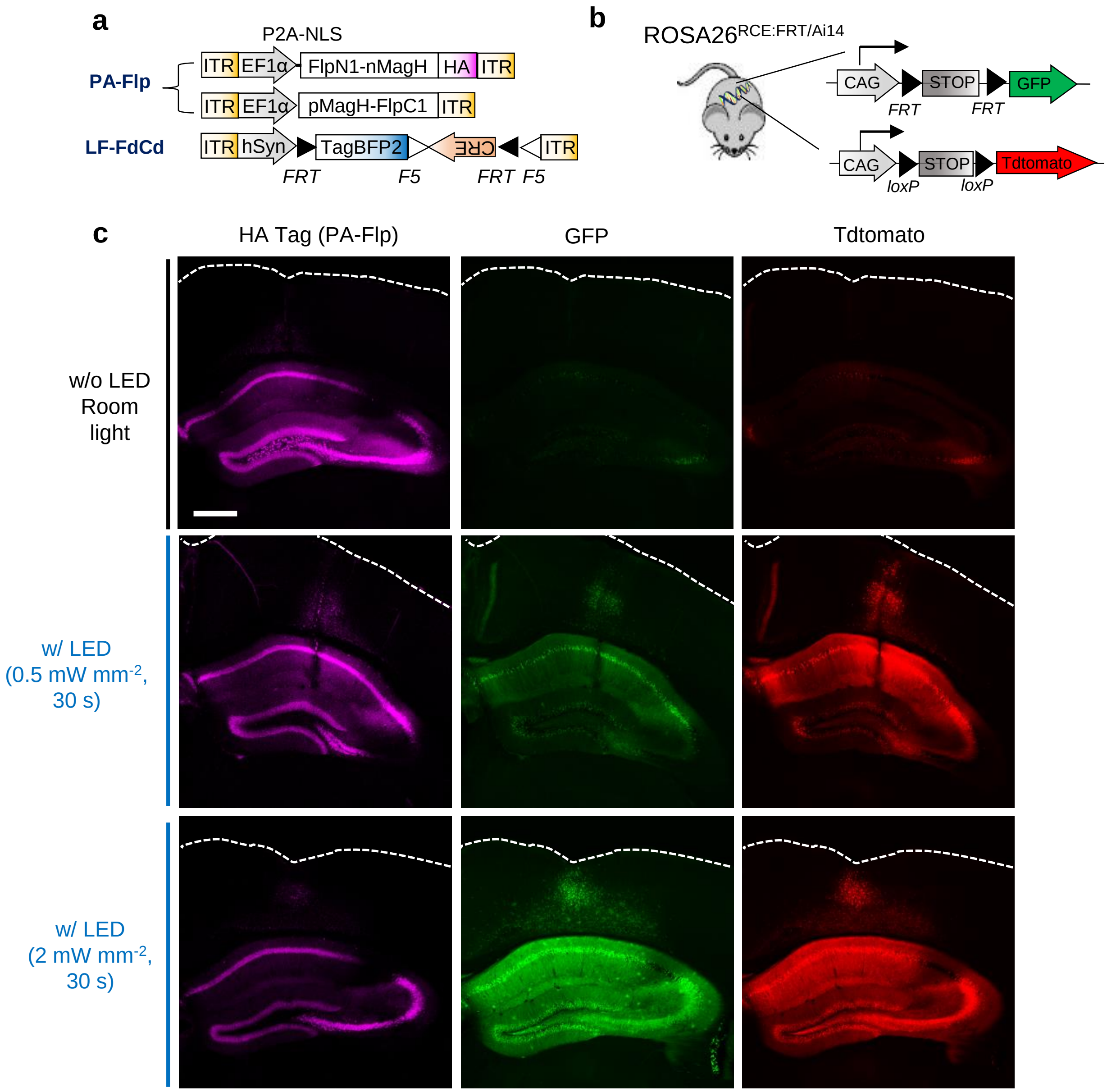

Supplementary Figure 12. Verification of the PA-FdCre system in the hippocampus using noninvasive LED delivery.

(a) Components of PA-Flp and LF-FdCd (same as in **Fig. 3a**).
(b) ROSA26$^{RCE:FRT/Ai14}$ mice (generation scheme shown in **Fig. 3b**).
(c) Fluorescence images of the hippocampus of 8–12-wk-old ROSA26$^{RCE:FRT/Ai14}$ mice infected with a mixture of AAVs carrying PA-Flp and LF-FdCd, obtained following illumination with the indicated intensities of noninvasive LED light for 30 s. w/ LED: with LED, w/o LED: without LED in room light. Scale bar: 500 μm.

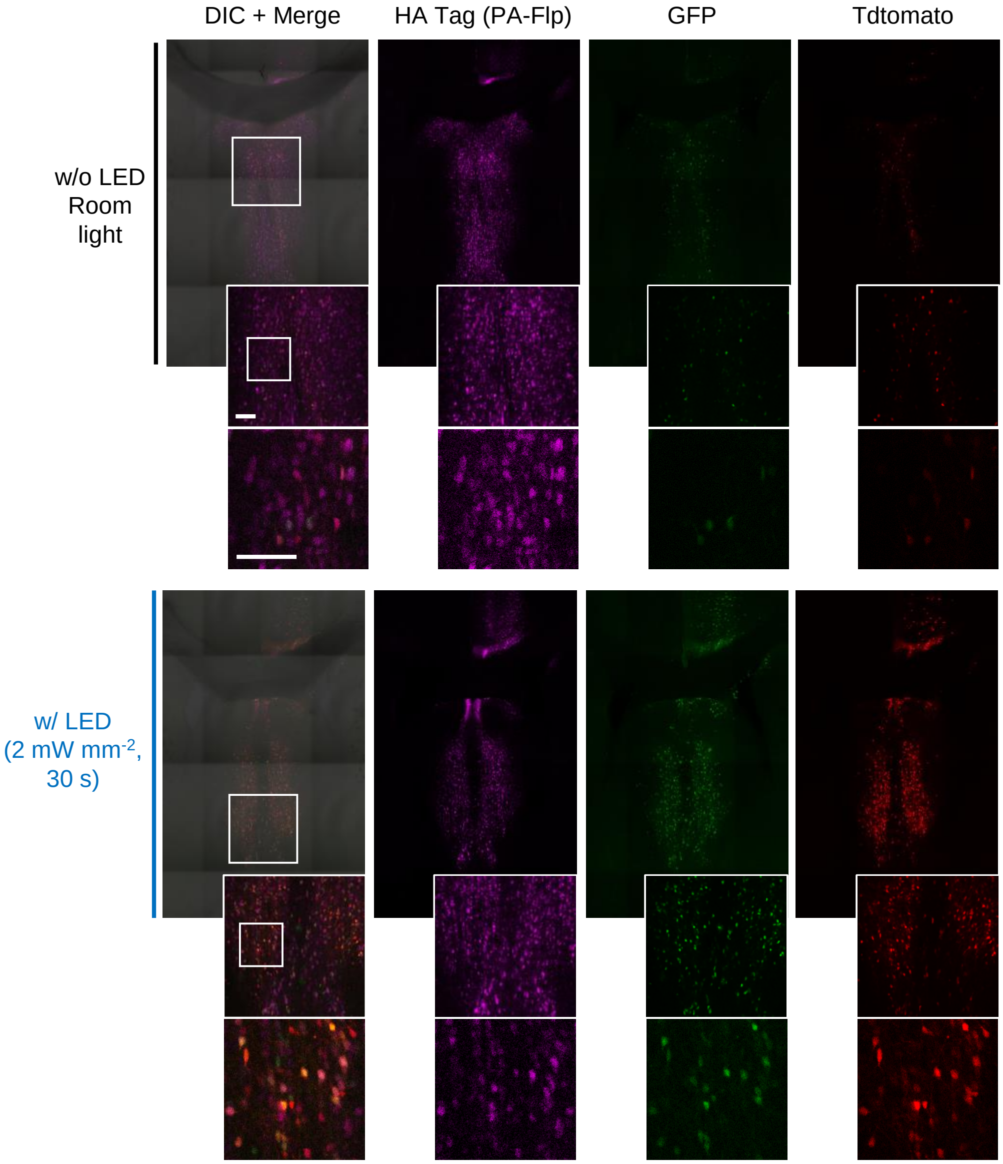

Supplementary Figure 13. Verification of the PA-FdCre system in the MS using noninvasive LED delivery.

Fluorescence images of the MS of 8-wk-old ROSA26$^{RCE:FRT/Ai14}$ mice infected with a mixture of AAVs carrying PA-Flp and LF-FdCd, obtained following noninvasive LED illumination (2 mW mm^{-2}, 30 s) or no illumination. Images below show enlargements of each white-boxed region. w/ LED: with LED, w/o LED: without LED in room light. Scale bar: 100 µm.

Supplementary Note 1

pAAV-EF1α::mCherry-FlpN-nMagHigh

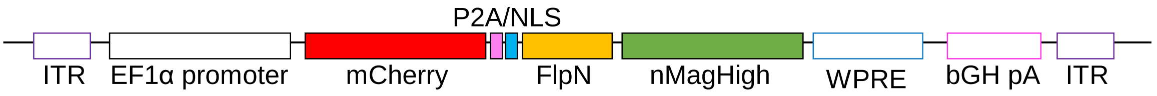

DNA sequence

CCTGCAGGCAGCTGCGCGCTCGCTCGCTCACTGAGGCCGCCCGGGCAAAGCCCGGGCGTCGGGCGACCTTTGGTCGCCCGGCCTCAGTGAGCGAGCGAG **ITR**
CGCGCAGAGAGGGAGTGGCCAACTCCATCACTAGGGGTTCCTGCGGCCGCACGCGTAAGCTTTGCAAAGATGGATAAAGTTTTAAACAGAGAGGAATCTT
TGCAGCTAATGGACCTTCTAGGTCTTGAAAGGAGTGGGAATTGGCTCCGGTGCCCGTCAGTGGGCAGAGCGCACATCGCCCACAGTCCCCGAGAAGTTGG
GGGGAGGGGTCGGCAATTGAACCGGTGCCTAGAGAAGGTGGCGCGGGGTAAACTGGGAAAGTGATGTCGTGTACTGGCTCCGCCTTTTTCCCGAGGGTG
GGGGAGAACCGTATATAAGTGCAGTAGTCGCCGTGAACGTTCTTTTTCGCAACGGGTTTGCCGCCAGAACACAGGTAAGTGCCGTGTGTGGTTCCCGCGG
GCCTGGCCTCTTTACGGGTTATGGCCCTTGCGTGCCTTGAATTACTTCCACTGGCTGCAGTACGTGATTCTTGATCCCGAGCTTCGGGTTGGAAGTGGGTGG
GAGAGTTCGAGGCCTTGCGCTTAAGGAGCCCCTTCGCCTCGTGCTTGAGTTGAGGCCTGGCCTGGGCGCTGGGGCCGCCGCGTGCGAATCTGGTGGCACC
TTCGCGCCTGTCTCGCTGCTTTCGATAAGTCTCTAGCCATTTAAAATTTTTGATGACCTGCTGCGACGCTTTTTTTCTGGCAAGATAGTCTTGTAAATGCGGG **EF1α**
CCAAGATCTGCACACTGGTATTTCGGTTTTTGGGGCCGCGGGCGGCGACGGGGCCCGTGCGTCCCAGCGCACATGTTCGGCGAGGCGGGGCCTGCGAGC **promoter**
GCGGCCACCGAGAATCGGACGGGGGTAGTCTCAAGCTGGCCGGCCTGCTCTGGTGCCTGGCCTCGCGCCGCCGTGTATCGCCCCGCCCTGGGCGGCAAG
GCTGGCCCGGTCGGCACCAGTTGCGTGAGCGGAAAGATGGCCGCTTCCCGGCCCTGCTGCAGGGAGCTCAAAATGGAGGACGCGGCGCTCGGGAGAGC
GGGCGGGTGAGTCACCCACACAAAGGAAAAGGGCCTTTCCGTCCTCAGCCGTCGCTTCATGTGACTCCACGGAGTACCGGGCGCCGTCCAGGCACCTCGA
TTAGTTCTCGAGCTTTTGGAGTACGTCGTCTTTAGGTTGGGGGGAGGGGTTTTATGCGATGGAGTTTCCCCACACTGAGTGGGTGGAGACTGAAGTTAGG
CCAGCTTGGCACTTGATGTAATTCTCCTTGGAATTTGCCCTTTTTGAGTTTGGATCTTGGTTCATTCTCAAGCCTCAGACAGTGGTTCAAAGTTTTTTTCTTCC
ATTTCAGGTGTCGTGAGGTACCGGATCCTCTAGAGTCGACTCCGGAACCATGGTGAGCAAGGGCGAGGAGGATAACATGGCCATCATCAAGGAGTTCATG
CGCTTCAAGGTGCACATGGAGGGCTCCGTGAACGGCCACGAGTTCGAGATCGAGGGCGAGGGCGAGGGCCGCCCCTACGAGGGCACCCAGACCGCCAA
GCTGAAGGTGACCAAGGGTGGCCCCCTGCCCTTCGCCTGGGACATCCTGTCCCCTCAGTTCATGTACGGCTCCAAGGCCTACGTGAAGCACCCCGCCGACA
TCCCCGACTACTTGAAGCTGTCCTTCCCCGAGGGCTTCAAGTGGGAGCGCGTGATGAACTTCGAGGACGGCGGCGTGGTGACCGTGACCCAGGACTCCTC **mCherry**
CCTGCAGGACGGCGAGTTCATCTACAAGGTGAAGCTGCGCGGCACCAACTTCCCCTCCGACGGCCCCGTAATGCAGAAGAAGACCATGGGCTGGGAGGC
CTCCTCCGAGCGGATGTACCCCGAGGACGGCGCCCTGAAGGGCGAGATCAAGCAGAGGCTGAAGCTGAAGGACGGCGGCCACTACGACGCTGAGGTCA
AGACCACCTACAAGGCCAAGAAGCCCGTGCAGCTGCCCGGCGCCTACAACGTCAACATCAAGTTGGACATCACCTCCCACAACGAGGACTACACCATCGT
GGAACAGTACGAACGCGCCGAGGGCCGCCACTCCACCGGCGGCATGGACGAGCTGTACAAGGGATCCGGCGCAACAAACTTCTCTCTGCTGAAACAAGC **P2A** / **NLS**
CGGAGATGTCGAAGAGAATCCTGGACCGCCCAAGAAGAAGAGGAAGGTGAGCCAGTTCGACATCCTGTGCAAGACCCCCCCCAAGGTGCTGGTGCGGCA **FlpN**
GTTCGTGGAGAGATTCGAGAGGCCCAGCGGTGGAGGCGGTTCAGGCGGAGGTGGCAGCGGCGGTGGCGGATCGCACACCCTGTACGCCCCCGGCGGCT **nMagHigh**
ACGACATCATGGGCTACCTGGACCAGATCGGCAACAGACCCAACCCCCAGGTGGAGCTGGGCCCCGTGGACACCAGCTGCGCCCTGATCCTGTGCGACCT
GAAGCAGAAGGACACCCCCATCGTGTACGCCAGCGAGGCCTTCCTGTATATGACCGGCTACAGCAACGCCGAGGTGCTGGGCAGAAACTGCAGATTCCTG
CAGAGCCCCGACGGCATGGTGAAGCCCAAGAGCACCAGAAAGTACGTGGACAGCAACACCATCAACACCATCAGAAAGGCCATCGACAGAAACGCCGAG
GTGCAGGTGGAGGTGGTGAACTTCAAGAAGAACGGCCAGAGATTCGTGAACTTCCTGACCATCATCCCCGTGAGAGACGAGACCGGCGAGTACAGATAC
AGCATGGGCTTCCAGTGCGAGACCGAGTAAGAATTCGATATCAAGCTTATCGATAATCAACCTCTGGATTACAAAATTTGTGAAAGATTGACTGGTATTCTT
AACTATGTTGCTCCTTTTACGCTATGTGGATACGCTGCTTTAATGCCTTTGTATCATGCTATTGCTTCCCGTATGGCTTTCATTTTCTCCTCCTTGTATAAATCC
TGGTTGCTGTCTCTTTATGAGGAGTTGTGGCCCGTTGTCAGGCAACGTGGCGTGGTGTGCACTGTGTTTGCTGACGCAACCCCCACTGGTTGGGGCATTGC **WPRE**
CACCACCTGTCAGCTCCTTTCCGGGACTTTCGCTTTCCCCCTCCCTATTGCCACGGCGGAACTCATCGCCGCCTGCCTTGCCCGCTGCTGGACAGGGGCTCG
GCTGTTGGGCACTGACAATTCCGTGGTGTTGTCGGGGAAATCATCGTCCTTTCCTTGGCTGCTCGCCTATGTTGCCACCTGGATTCTGCGCGGGACGTCCTT
CTGCTACGTCCCTTCGGCCCTCAATCCAGCGGACCTTCCTTCCCGCGGCCTGCTGCCGGCTCTGCGGCCTCTTCCGCGTCTTCGCCTTCGCCCTCAGACGAGT
CGGATCTCCCTTTGGGCCGCCTCCCCGCATCGATACCGAGCGCTGCTCGAGAGATCTACGGGTGGCATCCCTGTGACCCCTCCCCAGTGCCTCTCCTGGCCC
TGGAAGTTGCCACTCCAGTGCCCACCAGCCTTGTCCTAATAAAATTAAGTTGCATCATTTTGTCTGACTAGGTGTCCTTCTATAATATTATGGGGTGGAGGG
GGGTGGTATGGAGCAAGGGGCAAGTTGGGAAGACAACCTGTAGGGCCTGCGGGGTCTATTGGGAACCAAGCTGGAGTGCAGTGGCACAATCTTGGCTC **bGH pA**
ACTGCAATCTCCGCCTCCTGGGTTCAAGCGATTCTCCTGCCTCAGCCTCCCGAGTTGTTGGGATTCCAGGCATGCATGACCAGGCTCAGCTAATTTTTGTTTT
TTTGGTAGAGACGGGGTTTCACCATATTGGCCAGGCTGGTCTCCAACTCCTAATCTCAGGTGATCTACCCACCTTGGCCTCCCAAATTGCTGGGATTACAGG
CGTGAACCACTGCTCCCTTCCCTGTCCTTCTGATTTTGTAGGTAACCACGTGCGGACCGAGCGGCCGCAGGAACCCCTAGTGATGGAGTTGGCCACTCCCTC **ITR**
TCTGCGCGCTCGCTCGCTCACTGAGGCCGGGCGACCAAAGGTCGCCCGACGCCCGGGCTTTGCCCGGGCGGCCTCAGTGAGCGAGCGAGCGCGCAGCTG
CCTGCAGG

Amino acids sequence

MVSKGEEDNMAIIKEFMRFKVHMEGSVNGHEFEIEGEGEGRPYEGTQTAKLKVTKGGPLPFAWDILSPQFMYGSKAYVKHPADIPDYLKLSFPEGFKWERVMN
mCherry

FEDGGVVTVTQDSSLQDGEFIYKVKLRGTNFPSDGPVMQKKTMGWEASSERMYPEDGALKGEIKQRLKLKDGGHYDAEVKTTYKAKKPVQLPGAYNVNIKLDIT

SHNEDYTIVEQYERAEGRHSTGGMDELYKGSGATNFSLLKQAGDVEENPGPPKKKRKVSQFDILCKTPPKVLVRQFVERFERPSGGGGSGGGGSGGGGSHTLYA
P2A **NLS** **FlpN** **GS linker**

PGGYDIMGYLDQIGNRPNPQVELGPVDTSCALILCDLKQKDTPIVYASEAFLYMTGYSNAEVLGRNCRFLQSPDGMVKPKSTRKYVDSNTINTIRKAIDRNAEVQV
nMagHigh

EVVNFKKNGQRFVNFLTIIPVRDETGEYRYSMGFQCETE*

pAAV-EF1α::pMagHigh-FlpC

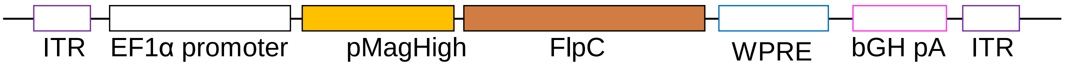

DNA sequence

CCTGCAGGCAGCTGCGCGCTCGCTCGCTCACTGAGGCCGCCCGGGCAAAGCCCGGGCGTCGGGCGACCTTTGGTCGCCCGGCCTCAGTGAGCGAGCGAG **ITR**
CGCGCAGAGAGGGAGTGGCCAACTCCATCACTAGGGGTTCCTGCGGCCGCACGCGTAAGCTTTGCAAAGATGGATAAAGTTTTAAACAGAGAGGAATCTT
TGCAGCTAATGGACCTTCTAGGTCTTGAAAGGAGTGGGAATTGGCTCCGGTGCCCGTCAGTGGGCAGAGCGCACATCGCCCACAGTCCCCGAGAAGTTGG
GGGGAGGGGTCGGCAATTGAACCGGTGCCTAGAGAAGGTGGCGCGGGGTAAACTGGGAAAGTGATGTCGTGTACTGGCTCCGCCTTTTTCCCGAGGGTG
GGGGAGAACCGTATATAAGTGCAGTAGTCGCCGTGAACGTTCTTTTTCGCAACGGGTTTGCCGCCAGAACACAGGTAAGTGCCGTGTGTGGTTCCCGCGG
GCCTGGCCTCTTTACGGGTTATGGCCCTTGCGTGCCTTGAATTACTTCCACTGGCTGCAGTACGTGATTCTTGATCCCGAGCTTCGGGTTGGAAGTGGGTGG
GAGAGTTCGAGGCCTTGCGCTTAAGGAGCCCCTTCGCCTCGTGCTTGAGTTGAGGCCTGGCCTGGGCGCTGGGGCCGCCGCGTGCGAATCTGGTGGCACC
TTCGCGCCTGTCTCGCTGCTTTCGATAAGTCTCTAGCCATTTAAAATTTTTGATGACCTGCTGCGACGCTTTTTTTCTGGCAAGATAGTCTTGTAAATGCGGG **EF1α**
CCAAGATCTGCACACTGGTATTTCGGTTTTTGGGGCCGCGGGCGGCGACGGGGCCCGTGCGTCCCAGCGCACATGTTCGGCGAGGCGGGGCCTGCGAGC **promoter**
GCGGCCACCGAGAATCGGACGGGGGTAGTCTCAAGCTGGCCGGCCTGCTCTGGTGCCTGGCCTCGCGCCGCCGTGTATCGCCCCGCCCTGGGCGGCAAG
GCTGGCCCGGTCGGCACCAGTTGCGTGAGCGGAAAGATGGCCGCTTCCCGGCCCTGCTGCAGGGAGCTCAAAATGGAGGACGCGGCGCTCGGGAGAGC
GGGCGGGTGAGTCACCCACACAAAGGAAAAGGGCCTTTCCGTCCTCAGCCGTCGCTTCATGTGACTCCACGGAGTACCGGGCGCCGTCCAGGCACCTCGA
TTAGTTCTCGAGCTTTTGGAGTACGTCGTCTTTAGGTTGGGGGGAGGGGTTTTATGCGATGGAGTTTCCCCACACTGAGTGGGTGGAGACTGAAGTTAGG
CCAGCTTGGCACTTGATGTAATTCTCCTTGGAATTTGCCCTTTTTGAGTTTGGATCTTGGTTCATTCTCAAGCCTCAGACAGTGGTTCAAAGTTTTTTTCTTCC
ATTTCAGGTGTCGTGAGGTACCGGATCCTCTAGAGTCGACTCCGGAACCATGCACACCCTGTACGCCCCCGGCGGCTACGACATCATGGGCTACCTGAGAC
AGATCAGAAACAGACCCAACCCCCAGGTGGAGCTGGGCCCCGTGGACACCAGCTGCGCCCTGATCCTGTGCGACCTGAAGCAGAAGGACACCCCCATCGT
GTACGCCAGCGAGGCCTTCCTGTATATGACCGGCTACAGCAACGCCGAGGTGCTGGGCAGAAACTGCAGATTCCTGCAGAGCCCCGACGGCATGGTGAA **pMagHigh**
GCCCAAGAGCACCAGAAAGTACGTGGACAGCAACACCATCAACACCATCAGAAAGGCCATCGACAGAAACGCCGAGGTGCAGGTGGAGGTGGTGAACTT
CAAGAAGAACGGCCAGAGATTCGTGAACTTCCTGACCATCATCCCCGTGAGAGACGAGACCGGCGAGTACAGATACAGCATGGGCTTCCAGTGCGAGACC
GAGGGTGGAGGCGGTTCAGGCGGAGGTGGCAGCGGCGGTGGCGGATCGGGCGAGAAGATCGCCAGCTGTGCCGCCGAGCTGACCTACCTGTGCTGGAT
GATCACCCACAACGGCACCGCCATCAAGAGGGCCACCTTCATGAGCTACAACACCATCATCAGCAACAGCCTGAGCTTCGACATCGTGAACAAGAGCCTGC
AGTTCAAGTACAAGACCCAGAAGGCCACCATCCTGGAGGCCAGCCTGAAGAAGCTGATCCCCGCCTGGGAGTTCACCATCATCCCTTACAACGGCCAGAA
GCACCAGAGCGACATCACCGACATCGTGTCCAGCCTGCAGCTGCAGTTCGAGAGCAGCGAGGAGGCCGACAAGGGCAACAGCCACAGCAAGAAGATGCT
GAAGGCCCTGCTGTCCGAGGGCGAGAGCATCTGGGAGATCACCGAGAAGATCCTGAACAGCTTCGAGTACACCAGCAGGTTCACCAAGACCAAGACCCT
GTACCAGTTCCTGTTCCTGGCCACATTCATCAACTGCGGCAGGTTCAGCGACATCAAGAACGTGGACCCCAAGAGCTTCAAGCTGGTGCAGAACAAGTACC **FlpC**
TGGGCGTGATCATTCAGTGCCTGGTGACCGAGACCAAGACAAGCGTGTCCAGGCACATCTACTTTTTCAGCGCCAGAGGCAGGATCGACCCCCTGGTGTA
CCTGGACGAGTTCCTGAGGAACAGCGAGCCCGTGCTGAAGAGAGTGAACAGGACCGGCAACAGCAGCAGCAACAAGCAGGAGTACCAGCTGCTGAAGG
ACAACCTGGTGCGCAGCTACAACAAGGCCCTGAAGAAGAACGCCCCCTACCCCATCTTCGCTATCAAGAACGGCCCTAAGAGCCACATCGGCAGGCACCT
GATGACCAGCTTTCTGAGCATGAAGGGCCTGACCGAGCTGACAAACGTGGTGGGCAACTGGAGCGACAAGAGGGCCTCCGCCGTGGCCAGGACCACCTA
CACCCACCAGATCACCGCCATCCCCGACCACTACTTCGCCCTGGTGTCCAGGTACTACGCCTACGACCCCATCAGCAAGGAGATGATCGCCCTGAAGGACG
AGACCAACCCCATCGAGGAGTGGCAGCACATCGAGCAGCTGAAGGGCAGCGCCGAGGGCAGCATCAGATACCCCGCCTGGAACGGCATCATCAGCCAGG
AGGTGCTGGACTACCTGAGCAGCTACATCAACAGGCGGATCTAAGAATTCGATATCAAGCTTATCGATAATCAACCTCTGGATTACAAAATTTGTGAAAGA
TTGACTGGTATTCTTAACTATGTTGCTCCTTTTACGCTATGTGGATACGCTGCTTTAATGCCTTTGTATCATGCTATTGCTTCCCGTATGGCTTTCATTTTCTCC
TCCTTGTATAAATCCTGGTTGCTGTCTCTTTATGAGGAGTTGTGGCCCGTTGTCAGGCAACGTGGCGTGGTGTGCACTGTGTTTGCTGACGCAACCCCCACT **WPRE**
GGTTGGGGCATTGCCACCACCTGTCAGCTCCTTTCCGGGACTTTCGCTTTCCCCCTCCCTATTGCCACGGCGGAACTCATCGCCGCCTGCCTTGCCCGCTGCT
GGACAGGGGCTCGGCTGTTGGGCACTGACAATTCCGTGGTGTTGTCGGGGAAATCATCGTCCTTTCCTTGGCTGCTCGCCTATGTTGCCACCTGGATTCTGC
GCGGGACGTCCTTCTGCTACGTCCCTTCGGCCCTCAATCCAGCGGACCTTCCTTCCCGCGGCCTGCTGCCGGCTCTGCGGCCTCTTCCGCGTCTTCGCCTTCG
CCCTCAGACGAGTCGGATCTCCCTTTGGGCCGCCTCCCCGCATCGATACCGAGCGCTGCTCGAGAGATCTACGGGTGGCATCCCTGTGACCCCTCCCCAGT
GCCTCTCCTGGCCCTGGAAGTTGCCACTCCAGTGCCCACCAGCCTTGTCCTAATAAAATTAAGTTGCATCATTTTGTCTGACTAGGTGTCCTTCTATAATATT
ATGGGGTGGAGGGGGGTGGTATGGAGCAAGGGGCAAGTTGGGAAGACAACCTGTAGGGCCTGCGGGGTCTATTGGGAACCAAGCTGGAGTGCAGTGG **bGH pA**
CACAATCTTGGCTCACTGCAATCTCCGCCTCCTGGGTTCAAGCGATTCTCCTGCCTCAGCCTCCCGAGTTGTTGGGATTCCAGGCATGCATGACCAGGCTCA
GCTAATTTTTGTTTTTTTGGTAGAGACGGGGTTTCACCATATTGGCCAGGCTGGTCTCCAACTCCTAATCTCAGGTGATCTACCCACCTTGGCCTCCCAAATT
GCTGGGATTACAGGCGTGAACCACTGCTCCCTTCCCTGTCCTTCTGATTTTGTAGGTAACCACGTGCGGACCGAGCGGCCGCAGGAACCCCTAGTGATGGA
GTTGGCCACTCCCTCTCTGCGCGCTCGCTCGCTCACTGAGGCCGGGCGACCAAAGGTCGCCCGACGCCCGGGCTTTGCCCGGGCGGCCTCAGTGAGCGAG **ITR**
CGAGCGCGCAGCTGCCTGCAGG

Amino acids sequence

MHTLYAPGGYDIMGYLRQIRNRPNPQVELGPVDTSCALILCDLKQKDTPIVYASEAFLYMTGYSNAEVLGRNCRFLQSPDGMVKPKSTRKYVD

pMagHigh

SNTINTIRKAIDRNAEVQVEVVNFKKNGQRFVNFLTIIPVRDETGEYRYSMGFQCETEGGGGSGGGGSGGGGSGEKIASCAAELTYLCWMITH

GS linker

NGTAIKRATFMSYNTIISNSLSFDIVNKSLQFKYKTQKATILEASLKKLIPAWEFTIIPYNGQKHQSDITDIVSSLQLQFESSEEADKGNSHSKKMLK

ALLSEGESIWEITEKILNSFEYTSRFTKTKTLYQFLFLATFINCGRFSDIKNVDPKSFKLVQNKYLGVIIQCLVTETKTSVSRHIYFFSARGRIDPLVYLD

EFLRNSEPVLKRVNRTGNSSSNKQEYQLLKDNLVRSYNKALKKNAPYPIFAIKNGPKSHIGRHLMTSFLSMKGLTELTNVVGNWSDKRASAVAR

TTYTHQITAIPDHYFALVSRYYAYDPISKEMIALKDETNPIEEWQHIEQLKGSAEGSIRYPAWNGIISQEVLDYLSSYINRRI*

FlpC

pAAV-hSYN::LF-FdCd

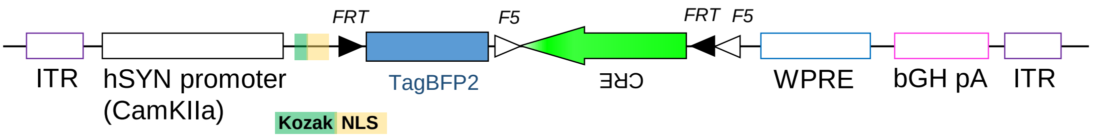

DNA sequence

CCTGCAGGCAGCTGCGCGCTCGCTCGCTCACTGAGGCCGCCCGGGCGTCGGGCGACCTTTGGTCGCCCGGCCTCAGTGAGCGAGCGAGCGCGCAGAGAG **ITR**
GGAGTGGCCAACTCCATCACTAGGGGTTCCTGCGGCCGCACGCGTGTGTCTAGACTGCAGAGGGCCCTGCGTATGAGTGCAAGTGGGTTTTAGGACCAGG
ATGAGGCGGGGTGGGGGTGCCTACCTGACGACCGACCCCGACCCACTGGACAAGCACCCAACCCCCATTCCCCAAATTGCGCATCCCCTATCAGAGAGGG **hSYN**
GGAGGGGAAACAGGATGCGGCGAGGCGCGTGCGCACTGCCAGCTTCAGCACCGCGGACAGTGCCTTCGCCCCCGCCTGGCGGCGCGCGCCACCGCCGCC **promoter**
TCAGCACTGAAGGCGCGCTGACGTCACTCGCCGGTCCCCCGCAAACTCCCCTTCCCGGCCACCTTGGTCGCGTCCGCGCCGCCGCCGGCCCAGCCGGACCG
CACCACGCGAGGCGCGAGATAGGGGGGCACGGGCGCGACCATCTGCGCTGCGGCGCCGGCGACTCAGCGCTGCCTCAGTCTGCGGTGGGCAGCGGAGG **Kozak NLS**
AGTCGTGTCGTGCCTGAGAGCGCAGTCGAGAAGGTACCGGTGCCACCATGCCCAAGAAGAAGAGGAAGGTGCTTCGTCCGGAGAAGTTCCTATTCTCTAG **FRT**

AAAGTATAGGAACTTCACCATGGTGTCTAAGGGCGAAGAGCTGATTAAGGAGAACATGCACATGAAGCTGTACATGGAGGGCACCGTGGACAACCATCA
CTTCAAGTGCACATCCGAGGGCGAAGGCAAGCCCTACGAGGGCACCCAGACCATGAGAATCAAGGTGGTCGAGGGCGGCCCTCTCCCCTTCGCCTTCGAC
ATCCTGGCTACTAGCTTCCTCTACGGCAGCAAGACCTTCATCAACCACACCCAGGGCATCCCCGACTTCTTCAAGCAGTCCTTCCCTGAGGGCTTCACATGG
GAGAGAGTCACCACATACGAAGACGGGGGCGTGCTGACCGCTACCCAGGACACCAGCCTCCAGGACGGCTGCCTCATCTACAACGTCAAGATCAGAGGG **mTagBFP2**
GTGAACTTCACATCCAACGGCCCTGTGATGCAGAAGAAAACACTCGGCTGGGAGGCCTTCACCGAGACGCTGTACCCCGCTGACGGCGGCCTGGAAGGCA
GAAACGACATGGCCCTGAAGCTCGTGGGCGGGAGCCATCTGATCGCAAACGCCAAGACCACATATAGATCCAAGAAACCCGCTAAGAACCTCAAGATGCC
TGGCGTCTACTATGTGGACTACAGACTGGAAAGAATCAAGGAGGCCAACAACGAGACCTACGTCGAGCAGCACGAGGTGGCAGTGGCCAGATACTGCGA
CCTCCCTAGCAAACTGGGGCACAAGCTTAATTAAGAAGTTCCTATTCTTCAAAAGGTATAGGAACTTCGGCGCGCCTTAGTCCCCATCCTCGAGCAGCCTCA **F5**
CCATGGCCCCAGTCTCAGAGTCCAGGTTTCTGATGTAGTTCATCACAATGTTCACATTGGTCCAGCCACCAGCCTGCATGATTTCAGGGATGGACACACCAG
CCCTGGCCATGTCCCTGGCAGCACCCACTCTGGCAGAGTGGCCAGACCAGGCCAGGTATCTCTGCCCAGAGTCATCCTTGGCACCATAGATCAGGCGGTG
GGTGGCCTCAAAGATCCCTTCCAGGGCCCGGGTGGACAGTTGGGAGGTGGCAGAAGGGGCAGCCACACCATTCTTTCTGACCCGGCAGAACAGGTAGTT
GTTGGGGTCATCAGCCACACCAGACACAGAGATCCATCTCTCCACCAGCTTGGTAACCCCCAGGGACAGGGCCTTCTCCACACCAGCTGTGGACACCAGGG
TCTTGGTCCTGCCAATGTGGATCAGCATTCTCCCACCATCGGTGCGGGAGATGTCCTTCACTCTGATTCTGGCAATTTCGGCAATGCGCAGCAGGGTGTTGT **CRE**
AGGCAATGCCCAGGAAGGCCAGGTTCCTGATGTCCTGGCATCTGTCAGAGTTCTCCATCAGGGATCTGACTTGGTCAAAGTCAGTGCGTTCAAAGGCCAG
GGCCTGCTTGGCTCTCTCCCCAGCATCCACATTCTCCTTTCTGATTCTCCTCATCACCAGGGACACAGCATTGGAGTCAGAAGGGCGAGGCAGGCCAGATCT
CCTGTGCAGCATGTTGAGCTGGCCCAGGTGCTGTTGGATGGTCTTCACAGCCAGGCCTCTGGCTTGCAGGTACAGGAGGTAGTCCCTCACATCCTCAGGTT
CAGCAGGGAACCATTTCCTGTTGTTCAGCTTGCACCAGGCAGCCCAGGATCTGCACACAGACAGGAGCATCTTCCAGGTGTGTTCAGAGAAGGCCTGCCTG
TCCCTGAACAGGTCCAGCAGGTTCTTCCTGACTTCATCAGAGGTGCTAGCGAAGTTCCTATACTTTCTAGAGAATAGGAACTTCTTGCCTTAACCCATGGTG **FRT**
GCTCACTGTTATTCTTTAGACCATGGTGGCGAATGGTGCAAAGAGAAGTTCCTATACCTTTTGAAGAATAGGAACTTCGAATTCGATATCAAGCTTATCGAT **F5**
AATCAACCTCTGGATTACAAAATTTGTGAAAGATTGACTGGTATTCTTAACTATGTTGCTCCTTTTACGCTATGTGGATACGCTGCTTTAATGCCTTTGTATCA
TGCTATTGCTTCCCGTATGGCTTTCATTTTCTCCTCCTTGTATAAATCCTGGTTGCTGTCTCTTTATGAGGAGTTGTGGCCCGTTGTCAGGCAACGTGGCGTG
GTGTGCACTGTGTTTGCTGACGCAACCCCCACTGGTTGGGGCATTGCCACCACCTGTCAGCTCCTTTCCGGGACTTTCGCTTTCCCCCTCCCTATTGCCACGG **WPRE**
CGGAACTCATCGCCGCCTGCCTTGCCCGCTGCTGGACAGGGGCTCGGCTGTTGGGCACTGACAATTCCGTGGTGTTGTCGGGGAAATCATCGTCCTTTCCT
TGGCTGCTCGCCTGTGTTGCCACCTGGATTCTGCGCGGGACGTCCTTCTGCTACGTCCCTTCGGCCCTCAATCCAGCGGACCTTCCTTCCCGCGGCCTGCTGC
CGGCTCTGCGGCCTCTTCCGCGTCTTCGCCTTCGCCCTCAGACGAGTCGGATCTCCCTTTGGGCCGCCTCCCCGCATCGATACCGTCGACCTCGAGAGATCT
ACGGGTGGCATCCCTGTGACCCCTCCCCAGTGCCTCTCCTGGCCCTGGAAGTTGCCACTCCAGTGCCCACCAGCCTTGTCCTAATAAAATTAAGTTGCATCA
TTTTGTCTGACTAGGTGTCCTTCTATAATATTATGGGGTGGAGGGGGGTGGTATGGAGCAAGGGGCAAGTTGGGAAGACAACCTGTAGGGCCTGCGGGG **bGH pA**
TCTATTGGGAACCAAGCTGGAGTGCAGTGGCACAATCTTGGCTCACTGCAATCTCCGCCTCCTGGGTTCAAGCGATTCTCCTGCCTCAGCCTCCCGAGTTGT
TGGGATTCCAGGCATGCATGACCAGGCTCAGCTAATTTTTGTTTTTTTGGTAGAGACGGGGTTTCACCATATTGGCCAGGCTGGTCTCCAACTCCTAATCTC
AGGTGATCTACCCACCTTGGCCTCCCAAATTGCTGGGATTACAGGCGTGAACCACTGCTCCCTTCCCTGTCCTTCTGATTTTGTAGGTAACCACGTGCGGAC
CGAGCGGCCGCAGGAACCCCTAGTGATGGAGTTGGCCACTCCCTCTCTGCGCGCTCGCTCGCTCACTGAGGCCGGGCGACCAAAGGTCGCCCGACGCCCG **ITR**
GGCTTTGCCCGGGCGGCCTCAGTGAGCGAGCGAGCGCGCAGCTGCCTGCAGG
