# Supplementary material for: Noninvasive optical activation of Flp recombinase for genetic manipulation in deep mouse brain regions
Source: Nat Commun. 2019 Jan 18;10:314. doi: 10.1038/s41467-018-08282-8 (PMC6338782; doi:10.1038/s41467-018-08282-8)
Supplement: Supplementary file 3 — Reporting Summary [file 41467_2018_8282_MOESM3_ESM.pdf]

nature research

Corresponding author(s): *Double-blind peer review submissions: write DBPR and your manuscript number here instead of author names.*

Last updated by author(s): *YYYY-MM-DD*

Reporting Summary

Nature Research wishes to improve the reproducibility of the work that we publish. This form provides structure for consistency and transparency in reporting. For further information on Nature Research policies, see Authors & Referees and the Editorial Policy Checklist.

Statistics

For all statistical analyses, confirm that the following items are present in the figure legend, table legend, main text, or Methods section.

n/a	Confirmed	
[ ]	[x]	The exact sample size (*n*) for each experimental group/condition, given as a discrete number and unit of measurement
[ ]	[x]	A statement on whether measurements were taken from distinct samples or whether the same sample was measured repeatedly
[ ]	[x]	The statistical test(s) used AND whether they are one- or two-sided<br>*Only common tests should be described solely by name; describe more complex techniques in the Methods section.*
[x]	[ ]	A description of all covariates tested
[x]	[ ]	A description of any assumptions or corrections, such as tests of normality and adjustment for multiple comparisons
[ ]	[x]	A full description of the statistical parameters including central tendency (e.g. means) or other basic estimates (e.g. regression coefficient) AND variation (e.g. standard deviation) or associated estimates of uncertainty (e.g. confidence intervals)
[ ]	[x]	For null hypothesis testing, the test statistic (e.g. *F*, *t*, *r*) with confidence intervals, effect sizes, degrees of freedom and *P* value noted<br>*Give P values as exact values whenever suitable.*
[x]	[ ]	For Bayesian analysis, information on the choice of priors and Markov chain Monte Carlo settings
[x]	[ ]	For hierarchical and complex designs, identification of the appropriate level for tests and full reporting of outcomes
[x]	[ ]	Estimates of effect sizes (e.g. Cohen's *d*, Pearson's *r*), indicating how they were calculated

Our web collection on statistics for biologists contains articles on many of the points above.

Software and code

Policy information about availability of computer code

Data collection	NIS-element AR 64-bit version 4.10; Laboratory Imaging
Data analysis	NIS-element AR 64-bit version 4.10; Laboratory Imaging, MetaMorph software (version 7.8.1.0, MDS Analytical Technologies)

For manuscripts utilizing custom algorithms or software that are central to the research but not yet described in published literature, software must be made available to editors/reviewers. We strongly encourage code deposition in a community repository (e.g. GitHub). See the Nature Research guidelines for submitting code & software for further information.

Data

Policy information about availability of data

All manuscripts must include a data availability statement. This statement should provide the following information, where applicable:

- Accession codes, unique identifiers, or web links for publicly available datasets
- A list of figures that have associated raw data
- A description of any restrictions on data availability

The data supporting the findings of this study are available within the paper and its supplementary information files. Extra data are available from the corresponding author upon reasonable request.

Field-specific reporting

Please select the one below that is the best fit for your research. If you are not sure, read the appropriate sections before making your selection.

[x] Life sciences [ ] Behavioural & social sciences [ ] Ecological, evolutionary & environmental sciences

For a reference copy of the document with all sections, see nature.com/documents/nr-reporting-summary-flat.pdf

Life sciences study design

All studies must disclose on these points even when the disclosure is negative.

Sample size	The sample size was determined by allowable error size and accuracy, and resources.
Data exclusions	The imaged slice samples with excessive expression level measured by fluorescence intensity over 4,000 arbitrary unit (NIS-element AR 64-bit, Nikon imaging software) were excluded from Fig 3 e,f.
Replication	All measured data were analyzed by at least three times of independent trials except for Fig 1g,h. The data from Fig 1g,h was analyzed by one mouse brain sample to measure the GFP signals as indicated parameters. All results are reported as average of multiple experiments with their variance.
Randomization	The samples were randomly grouped.
Blinding	The investigators were blinded to group allocation during data collection and analysis.

Reporting for specific materials, systems and methods

We require information from authors about some types of materials, experimental systems and methods used in many studies. Here, indicate whether each material, system or method listed is relevant to your study. If you are not sure if a list item applies to your research, read the appropriate section before selecting a response.

Materials & experimental systems

n/a	Involved in the study	
☐	☒	Antibodies
☐	☒	Eukaryotic cell lines
☒	☐	Palaeontology
☐	☒	Animals and other organisms
☒	☐	Human research participants
☒	☐	Clinical data

Methods

n/a	Involved in the study	
☒	☐	ChIP-seq
☒	☐	Flow cytometry
☒	☐	MRI-based neuroimaging

Antibodies

Antibodies used	Anti-HA-Tag rabbit mAb (#3724, CST), anti-Cav3.1 (#ACC-021, Alomone Lab)
Validation	The specificity of Anti-HA-Tag has been confirmed by overexpressing HA-tagged protein at the rAAV delivered sites in sliced mouse brain. The Anti-Cav3.1 has been confirmed by endogenous expression patterns of Cav3.1 in cortex, thalamus or medial-septum of sliced mouse brain samples.

Eukaryotic cell lines

Policy information about cell lines

Cell line source(s)	HEK293T (from ATCC), Primary hippocampal culture neuron from E18 pregnant Sprague-Dawley female rats purchased from ORIENT BIO Inc.
Authentication	Commercially proved (ATCC), It gives high titers when used to produce Retrovirus, Lentivirus or AAV. It is a highly transfectable.
Mycoplasma contamination	The cell line was confirmed negative for mycoplasma contamination by PCR-based mycoplasma detection methods.
Commonly misidentified lines (See ICLAC register)	None

Animals and other organisms

Policy information about studies involving animals; ARRIVE guidelines recommended for reporting animal research

Laboratory animals	C57BL/6J, Male, 8-12 week-old.
Wild animals	The study did not involve wild animals

Field-collected samples	The study did not involve samples collected from the field
Ethics oversight	Animal care and experimental procedures followed the guidelines of the Institutional Animal Care and Use Committee of Institute for Basic Science.

Note that full information on the approval of the study protocol must also be provided in the manuscript.
